# Influence of personal resources on job satisfaction. A study among professionals in the inpatient care of children and adolescents

**DOI:** 10.1192/j.eurpsy.2022.776

**Published:** 2022-09-01

**Authors:** S. Haehnle, J.M. Fegert, U. Hoffmann

**Affiliations:** University Hospital Ulm, Child And Adolescent Psychiatry, Ulm, Germany

**Keywords:** job satisfaction, child and youth residential care, personal resources, self-efficacy

## Abstract

**Introduction:**

Professionals in the inpatient care of burdened children and adolescents are confronted with high demands in their daily work. The job satisfaction can be affected negatively, if these professionals do not have the necessary resources to carry out their work.

**Objectives:**

In a study as part of the accompanying research of an online course called “Trauma informed Care”, the connection between the personal resources action competence, emotional competence, self-efficacy and self-care and job satisfaction were investigated on a sample of N = 543 professionals working in the (inpatient) care of children and adolescents.

**Methods:**

In order to quantify the connections between the personal resources action competence, emotional competence, self-efficacy and self-care and job satisfaction, correlations and a multiple regression were calculated.

**Results:**

Moderate to strong correlations were identified between personal resources and job satisfaction among the professionals. The regression model revealed self-efficacy to be the most important predictor of job satisfaction. Self-care was also identified as an important predictor. Less importance could be ascribed to emotional competence. Action competence showed no effects in the regression model.

**Conclusions:**

The results indicate the importance of personal resources for job satisfaction an their targeted promotion in order to increase job satisfaction and thus counteract the tendency of fluctuation and shortage of professionals in the area of child and youth welfare.

**Disclosure:**

No significant relationships.

